# Common Affections of the Skin of the Perinæum and Adjacent Parts

**Published:** 1907-07-06

**Authors:** Malcolm Morris

**Affiliations:** Dermatologist to King Edward VII. Hospital, Surgeon to the Skin Department Seamen's Hospital, Greenwich


### Hospital Clinics.

### COMMON AFFECTIONS OF THE SKIN OF THE PERINÆUM AND ADJACENT PARTS.

By MALCOLM MORRIS, F.R.C.S. Ed., Dermatologist to King Edward VII. Hospital, Surgeon to the Skin Department Seamen's Hospital, Greenwich.

I propose to-day to speak of common affections of the skin of the perinæum, the external genitals, the folds of the groins, and the buttocks, or more generally of what may be called the "bathing drawers area." That region presents a number of special features which modify the appearance and course of evolution of the lesions characteristic of various diseases which affect the integument. The skin of the perinæum presents the puckered ridge of the raphé, with the wrinkled contractile scrotum, while about the anus it is arranged in countless tiny folds radiating from the orifice, each of which may be the hiding-place of irritating secretion, dirt, or ulceration. Around the margin of the anus and on the scrotum the skin is deeply pigmented. Further, this region is exposed to an exceptional amount of irritation from sweating, and, unless the most scrupulous precautions are used, also from urine and particles of fæcal matter. Added to these there is friction from clothes and from mutual chafing of surfaces in contact with each other in walking. Again, the presence of orifices (anus, vulva, meatus) at whose edges skin and mucous membrane meet, supplies conditions favourable for the development of certain diseases of the integument. The hairiness of the parts renders them peculiarly liable to invasion by micro-organisms, and the warmth and moisture of the region make it a kind of forcing-house for germs. Owing to these causes the diseases of the perinæum and adjacent parts require special treatment, or rather the special adaptation of ordinary remedies.

### The Commoner Skin Affections.

I may say here that rare forms of disease, such as vegetating follicular psoro-spermosis, do not fall within the purview of these lectures. Some diseases of the skin peculiar to the tropics are, however, occasionally met with in this country in persons who have lived abroad, and these will require to be touched upon. But it is the commoner affections with which we are more especially concerned. Though most of them may be looked upon—by those who have never suffered from them—as comparatively trivial ailments, they are well worthy of the attention of practitioners. Though they do not as a rule threaten life, they cause an amount of bodily and mental misery quite out of proportion to their pathological importance. Moreover, there is no part of the body in which the diagnosis and treatment of tegumentary affections are more difficult.

The diseases to be discussed may be arranged roughly in their order of frequency as follows: Intertrigo, eczema, contagious impetigo, boils, scabies, pediculosis, psoriasis, lichen planus, ringworm, tinea versicolor, herpes, pruritus (anus, vagina, etc.), and syphilitic lesions.

### General Points.

*Examination.*—First as to the examination of the patient. Lesions that supply the clue to the nature of the disease may easily be overlooked unless every fold, nook, and cranny is carefully explored. It is important, therefore, that the parts should be inspected in a good light. The examination should first be made from the front, special attention being directed to the folds of the groin; to the hypogastric folds if the patient be fat and to the genitals if there is any reason to suspect the presence of any lesion there. Next examine the back, the cleft between the nates, the perinæum, the anus, and if anything should point in these directions the penis or the vulva. Omission to make such a complete survey may lead to the point of origin of the disease remaining undetected. Critically examine whatever lesions may be present, differentiating the primary lesions characteristic of a given disease from those produced by scratching and secondary infection of the tracks of the nails. The exact point of origin should, if possible, be determined, and the patient should be asked to indicate the exact spot in which the trouble began. A history of previous skin affections, either in the region under investigation or in any other part of the body, should be sought for, as it will often furnish the only key to the situation. In the region under consideration the characteristic lesions of the various skin diseases are apt to have their identity quickly obscured by the friction of adjacent surfaces and by maceration. For example, in a case of psoriasis the lesions themselves may tell you nothing. But psoriasis is a symmetrical disease which usually begins on the elbows and knees and other parts of the extensor surfaces of the limbs. Therefore these parts should always be examined; if the marks of psoriasis are found, the nature of the disease in the perinæum, etc., is revealed. On the other hand, if unmistakable psoriatic lesions exist only in the cleft between the buttocks or anywhere in the "bathing drawers area," that fact is sufficient to prove that the disease in these situations is probably a remnant of a general attack.

### Age and Sex.

The age and, of course, the sex must be taken into account in making the diagnosis. In infants intertrigo, eczema, and congenital syphilis are the most common affections. In young people intertrigo, eczema, impetigo contagiosa, herpes genitalis, pediculosis ("crabs") are the most frequent. The advent of middle-age brings with it a tendency to certain skin diseases, secondary either to constitutional states or to affections of internal organs. The constitutional state may either be a predisposing or a directly causative factor. Thus chronic dyspepsia, gout, rheumatism, cardiac disease, albuminuria, and nervous disorders, from their injurious effect on the nutrition of the skin, or from the disturbance of the circulation which they cause, lower its resistance, or induce local conditions favourable for the development of disease. Then nervous excitability, without actual disease, has a strong predisposing influence. Diabetes, as is well known, is apt to produce cutaneous lesions on the genitals by the direct irritation of the sugar-loaded urine, and predisposes to boils and carbuncles.

Among the internal diseases occurring in middle-age which often give rise to cutaneous complications in the perinæal region, may be mentioned hemiplegia. Unless the patient is kept absolutely clean the irritation of the urine and fæces is sure to cause inflammatory lesions which, owing to the low vitality of the patient, are apt to take on unhealthy action. Kidney disease and piles are often associated with pruritus ani, and eczema is a constant accompaniment of enlargement of the prostate. In women the change of life, owing to the circulatory disturbance which is one of its principal features, is frequently accompanied by pruritus of the vulva and by local skin lesions caused by scratching. Vaginal discharge is a frequent cause of similar troubles.

In old age, when the nutrition of the skin has generally fallen below the standard of vigorous health, pruritus in various forms is particularly common. Eczema is also frequent.

### Mode of Life.

Inquiry must be made as to the patient's mode of life—his food and drink; his clothing, especially that which is worn next the skin; whether he has lived in the tropics. His temperament and disposition must be studied, as the "personal equation" has to be reckoned with in estimating the value of the evidence he gives about himself. Persons of a nervous temper not only complain more, but actually suffer more, than others. But it may be well to warn the practitioner not to make light of pruritus and other conditions in which the subjective sensations may appear to be exaggerated, because there is not enough visible mischief to account for them. Pruritus of the anus and of the vulva, by the continuous irritation which they produce and the consequent sleeplessness, often cause nervous prostration which may ultimately endanger reason, and even lead to suicide. It is in neurotic subjects, and more especially women, that the drug habit is most common. As to this, the practitioner must inquire with particular care. Apart from their injurious effects in other directions, bromides, opium, cocaine, and some other narcotics may all give rise to eruptions of various kinds.

### General Principles of Treatment.

A few words as to the general principles of treatment. They are those which apply to skin disease of all kinds. Constitutional states must be dealt with by appropriate measures, but the treatment must be mainly local. The objects of local treatment may be summarised as follows:—(1) To soothe; (2) to disinfect and cleanse; (3) to set up reaction in order to destroy micro-organisms or to reduce hypertrophy of epidermis or corium; (4) to cause peeling of the horny layer. For these different purposes remedies are available which may be used in various forms. The following is a list of the means at our disposal:—(1) Baths; (2) soaps; (3) powders; (4) lotions; (5) ointments; (6) pastes; (7) creams; (8) gelatines; (9) varnishes; (10) plasters. I only enumerate these vehicles here.

As one of the difficulties of treating affections of the region we are considering is the keeping of dressings in position, this seems a fitting place to describe the plan I adopt for this purpose. Strips of linen steeped in lotion or covered with ointment should be applied. A bandage should then be put round the waist, to which one corner of a handkerchief should be attached. This should be split in two, and the ends passing on either side of the genitals should be taken up the back and fastened to the waist bandage behind.

### Intertrigo.

The most common of the diseases we have to consider is *Intertrigo.* This is an erythema caused by the chafing of opposed surfaces such as the folds of the groin, internal aspects of the thighs, the scrotum, the nates, or where the abdomen hangs over the pubic region. Obesity is a predisposing cause. The affection presents certain differences of character in infancy, in middle life, and in old age. In nurslings, the eruption generally corresponds to the parts that are in contact with the napkins. There is no exudation, but the epidermis is generally more or less macerated by sweat. In adults the ordinary seat is the side of the scrotum and the inner part of the thigh. The surface is red, glazed and hot; itching is usually very troublesome, sometimes almost maddening; and there may be severe pain. Intertrigo may come on acutely. The diagnosis is generally easy. The limitation of the erythema to the surfaces which have been exposed to chafing is at once suggestive of intertrigo. The only condition with which it is at all likely to be confounded is eczema, and from that affection it is readily distinguishable by the absence of the characteristic "weeping." In young children it is sometimes difficult to distinguish intertrigo from the erythematous eruption of congenital syphilis. The former, however, is, as a rule, limited to the parts covered by the napkins, whereas the latter spreads down the legs, often reaching to the heels and soles of the feet, while characteristic lesions, such as mucous tubercles, are to be found elsewhere.

### Treatment.

The essential point in the treatment of intertrigo is to prevent the chafing. In infants, the parts must be kept dry by changing the napkins as often as may be required, and by careful cleansing. Irritative lesions are most apt to occur when the urine or fæces are not normal. In such case calomel may with advantage be given in doses of one-fifth of a grain. If worms are present appropriate treatment must be used. The opposing surfaces must be kept apart by means of small pads of lint or cotton wool placed not on the diseased area, but above and below it, or by the interposition of a bag made of old, used linen or other material not too thick; the pieces should be evenly cut and sewn together, one edge being left open so that the bag may be partly filled with a powder such as one of the following:—

1. Oxide of zinc, 1 part to 3 parts of powdered rice, starch, maize, or kaolin.
2. Finely-powdered boric acid, 1 part to 3 parts of rice, starch, kaolin, or fuller's earth.

As decomposition of the secretions on the affected surfaces is likely to take place and make the irritation worse, the parts should frequently be washed with a solution of boracic acid (grs. 10 to 15 in one ounce of distilled water), carefully dried and afterwards dusted over with a protective powder. For this purpose either one of those just named, or salicylic acid 3 parts, powdered talc 87 parts, powdered starch 10 parts, may be used.

In the adult the first point to be decided is whether or not the patient should lie up. The decision of this question depends on the severity of the lesions. If there is mere sweat irritation and the subjective symptoms are not severe, he may be allowed to go about keeping the parts powdered, but he should not walk about much, and the affected surfaces should be kept cool with calamine lotion applied night and morning. Strict injunctions must be given against scratching, which, besides aggravating the condition, is apt to produce secondary lesions that may easily offer points of entry to pyogenic microbes.

### ECZEMA.

Eczema about the genitals is generally of the erythematous form, and the inflammation is most severe where there is friction between adjacent surfaces. The itching is so intense that the most determined will can scarcely keep the patient from scratching. The scrotum and penis often become greatly swollen, and the disease may extend over the perinæum, round the anus, into the cleft between the nates and over the buttocks, in the fold of which it may cause deep painful cracks; not infrequently it extends over the whole "bathing drawers area." A patient in this state cannot sit down or walk without the crusts and the inflamed skin beneath them giving way; painful cracks are thus produced. In the female things are still worse. The whole process is fanned into a fierce flame by continual chafing and the acrid discharges. Almost every variety of lesion that can be caused by acute inflammation, aggravated by scratching and urine, crusts and scabs from dried discharge and fissures may be present. Eczema of the anus is often associated with external piles; the skin is thickened, and there are often painful fissures. In eczema of the vulva the parts are greatly swollen and reddened, and so painful that contact with clothes or movement is acutely painful; the passage of urine causes scalding, and there is an offensive discharge. Both in this condition and in eczema of the anus there is severe itching, and the harrassing character of the affection gives a haggard expression to the countenance. In sucklings the nates and thighs are often the seat of eczematous lesions. These are frequently overlooked, as the mothers and nurses do not, in washing the babe, separate the parts sufficiently for fear of making it cry. What is conveniently, though perhaps unscientifically, known as "sweat eczema" occurs in adults. Excessive secretion of sweat makes the skin vulnerable to the action of the parasite or whatever the cause may be which produces eczema.

### Treatment.

In the treatment of eczema, constitutional must be combined with local measures. In acute cases the first indication is physiological rest. The patient should be kept in bed, and he should be told to move as little as possible. He should not be so warm as to promote sweating; therefore his own clothing should be light, and the bed should not be loaded with heavy coverings. The room should be kept at a moderate temperature not exceeding 60° F. An important element in the treatment is a nurse who can be trusted not to worry the patient by too much fussiness. Repose of mind is as necessary as bodily rest. The healthy action of the bowels and kidneys should be encouraged. The urine should be examined, as glycosuria is often present in fat patients, while in the thin, diabetes is not infrequent. This must be treated, as sugar in the urine is a serious aggravating circumstance, if it be not the actual cause of the skin affection. If the symptoms are very acute, milk diet should be enjoined, and throughout the illness the food should be of the simplest. In cases of average severity, no restrictions in the matter of food are necessary; abstinence from alcohol and coffee should, however, be insisted on, as both aggravate the symptoms. The cure in adults may be completed by change of air, not however at the seaside, and by a course of sulphur waters.

### The Value of Antimony.

If the local inflammation runs high, the internal administration of small doses of antimony will relieve the arterial tension.

If the constitution is sound I begin by giving 5 minims of antimony wine, repeating in one hour, and, if necessary, in two or three hours. After this it should be given three times a day for two, three, or four days. But antimony should not be given at all where there is much depression, and I may remind you here that arsenic is almost invariably contra-indicated in all these acute diseases of the skin.

When nervous symptoms are present small doses of opium or morphine associated with antimony are of service. Chloral, phenacetin, sulphonal, or veronal may be substituted according to indications. I know no circumstances connected with the treatment of this disease which taxes the practitioner's ingenuity more than the use of narcotics. We have first to consider idiosyncrasies. After more than 30 years' active practice I have to admit that I cannot state which is the best narcotic for the allaying of itching, or which best helps the patient to bear the torture of itching. I make it a rule to ask the patients about their experiences of narcotics before prescribing, and in this way I obtain some light as to whether the individual has any idiosyncrasies. Recently in consultation with a medical man I saw a patient who had been given a small dose of veronal to allay the irritation, with the result that for the first three nights the administration of 10 grains each night was followed by singular relief. On the fourth night veronal was taken as before but with the most disastrous results. A condition of coma was produced, and the drug had to be entirely suspended, as the patient became ill from veronal poisoning. Sulphonal was afterwards tried, but, as a rule it has not the power to produce sufficient amount of sleep to overcome the torture of itching. This patient suffered terribly because none of these other remedies could give him sleep. In some persons fairly large doses of chloride of calcium will be of assistance, and every now and then where narcotics fail large doses of a simple alkali sometimes render great service. Half a dram of bicarbonate of potash with peppermint-water administered the very last thing at night, sometimes produces great benefit. Great prostration calls for quinine, which may be combined with opium. When the discharge is very profuse the quinine may be more advantageously combined with belladonna.

### Local Measures.

In an acute case of discharging eczema, the parts must not be washed with soap; only rain-water or water that has been boiled should be used. Drying must be done very gently, as rough friction makes the condition worse. The calamine lotion is very soothing for the early stage of acute eczema. It may be made up as follows:—

| | |
|---|---|
| Oxide of zinc ... ... ... ... | 1 dram |
| Calamine ... ... ... ... ... | 2 drams |
| Lime-water ... ... ... ... | 2 oz. |
| Rose-water ... ... ... ... ... | 8 oz. |

When there is much discharge a weak solution of boric acid should be dabbed on with a wet cloth. When irritation is kept up by the catarrh of the bladder or pus in the urine, it is a matter of importance not to use injections of boric acid, or to give it internally. It aggravates the eczema. I have seen a great number of cases where this drug has been used and the irritation has been kept up outside by some reflected action in spite of the use of soothing applications. It may be taken as a rule of practice that there is a risk in using boric acid in the bladder when the skin is inflamed.

Parasiticide substances must at first be used tentatively. Beginning with weak preparations, the strength should be increased gradually so that irritation may be not caused. Precipitated sulphur may be applied on strips of linen, at first in the strength or gr. x. to one ounce of zinc ointment, increased according to tolerance. Resorcin may be used in the same way. Ichthyol, in addition to its microbicide properties, has a further value as a sedative. A solution or an ointment applied to the inflamed area allays irritation, causes contraction of the blood-vessels, and thus checks discharge. If crusts and scales have formed these must be removed, as a preliminary to the application of local remedies. They should first be softened with oil applied on strips of lint, or weak solutions of bicarbonate of soda. For the protection of the inflamed surface and for the relief of irritation one of the following applications may be used:—

| | |
|---|---|
| ℞ Zinci oxidi ... ... ... ... ... | ʒvj. |
| Lanolini ... ... ... ... ... | ʒij. |
| Ol. olivæ ... ... ... ... ... | ℥j. |
| Aquæ calcis ... ... ... ... ... | ℥j. |
| M. | |

| | |
|---|---|
| ℞ Aq. rosarum. ... ... ... ... | 10.0 |
| Ol. amygdal. ... ... ... ... | 10.0 |
| Ceræ albæ ... ... ... ... ... | 1.0 |
| Cetacei ... ... ... ... ... | 1.0 |

This may be used by itself as a cooling salve, or it may be made the excipient of antiseptic agents.

When discharge is no longer profuse, Unna's salve muslins may be used. They consist of muslin spread with a consistent layer of benzoated lard and wax; the lard may be replaced by vaseline or lanolin. These muslins may be made vehicles for carbolic acid, ichthyol, salicylic acid, sulphur, resorcin, etc.; they should be trimmed so as to fit the part to which they are to be applied. As salve muslins are expensive, a substitute can be made by spreading a stiff ointment on butter-cloth. For chronic patches left behind after the subsidence of an acute attack, plaster mulls are better adapted. There is also the zinc glycerine jelly or varnish, the advantage of which is that it can be applied to any part of the body so as to form a covering at once tight fitting and pliable; but this is only suitable to the legs and buttocks, and not to the folds. These applications, kept in position as described, will allow the patient to get about. But till the disease has wholly disappeared, exercise must be moderate, and the patient should lie up again at the first sign of a fresh eruption.

In pustular eczema, when there is much discharge, painting with a solution of ichthyol is effective; a strength of 1 to 8 of water may be used to begin with. For the deep painful cracks between the buttocks, painting with a strong solution of ichthyol (1 in 3-4) is useful. In eczema of the vagina the patient should be kept on her back, and the part plugged with lint steeped in lotio nigra or weak ichthyol solution (1 in 16). For moist lesions in nurslings oxide of zinc ʒii. with vaseline, lanolin, and rose-water, of each an ounce; or oxide of zinc 18 parts, carbonate of bismuth 2 parts, and vaseline 20 parts are useful applications.

### Impetigo Contagiosa.

Impetigo contagiosa is a locally infective disease caused by the inoculation of pus cocci. It begins as a crop of erythematous spots, on which vesicles form, whose contents soon become purulent; the pustules soon burst, and discharge a fluid that quickly dries up, forming yellowish scabs. A characteristic feature is the absence of hyperæmia round the scabs, which look as if they were stuck on the skin with gum. In the perinæum the friction of opposed surfaces favours rupture of the pustules and inoculation of their contents. The affected parts and the adjacent skin should be washed with a weak antiseptic such as boracic acid or carbolic lotion (1 in 100) as a measure both of cure and of prevention. Afterwards ung. hyd. ammon. should be applied, spread on linen and laid within the folds of the skin. Scratching should, as far as possible, be prevented. Weakly and ill-nourished subjects will be benefited by cod-liver oil and iron.

